# Competition explains limited attention and perceptual resources: implications for perceptual load and dilution theories

**DOI:** 10.3389/fpsyg.2013.00243

**Published:** 2013-05-10

**Authors:** Paige E. Scalf, Ana Torralbo, Evelina Tapia, Diane M. Beck

**Affiliations:** ^1^Department of Psychology, University of ArizonaTucson, AZ, USA; ^2^Institute of Cognitive Neuroscience, University College LondonLondon, UK; ^3^Department of Psychology, Beckman Institute, University of IllinoisUrbana, IL, USA

**Keywords:** perceptual load, competition, dilution, limited capacity, limited resources

## Abstract

Both perceptual load theory and dilution theory purport to explain when and why task-irrelevant information, or so-called distractors are processed. Central to both explanations is the notion of limited resources, although the theories differ in the precise way in which those limitations affect distractor processing. We have recently proposed a neurally plausible explanation of limited resources in which neural competition among stimuli hinders their representation in the brain. This view of limited capacity can also explain distractor processing, whereby the competitive interactions and bias imposed to resolve the competition determine the extent to which a distractor is processed. This idea is compatible with aspects of both perceptual load and dilution models of distractor processing, but also serves to highlight their differences. Here we review the evidence in favor of a biased competition view of limited resources and relate these ideas to both classic perceptual load theory and dilution theory.

## Introduction

“Everyone knows what attention is …it implies withdrawal from some things in order to deal effectively with others” (James, 1890). For over a century, psychologists have understood that the primary problem with attention is that we do not have enough of it; we simply cannot process and respond to all the information in the environment that may be relevant to our current task (e.g., Jersild, 1927; Cherry, 1953; Welford, 1957; Broadbent, 1958; Sperling, 1960; Eriksen and St. James, 1986; Pylyshyn and Storm, 1988; Raymond et al., 1992; Pashler, 1994); nor can we completely inhibit distracting information (e.g., Stroop, 1935; Treisman, 1960; Eriksen and Eriksen, 1974). More recent research has sought to understand the neural basis of our limited attentional capacity, and has revealed neural limits in our capacity to prioritize (e.g., Mecklinger et al., 2003), encode into working memory (e.g., Todd and Marois, 2004; Scalf et al., 2007, 2011b), and respond to task-relevant material (e.g., dual task interference Dux et al., 2006; Erickson et al., 2007).

The prevailing model and thus investigations of our limited capacity to attend to multiple items have focused on our limited attentional resources (e.g., Intriligator and Cavanagh, 2001; Lavie and Robertson, 2001; Mitchell and Cusack, 2008; Xu and Chun, 2009). These models have suggested that it is our limited ability to simultaneously direct attention to multiple stimuli that causes our limited capacity to respond to those items. They proceed from a “resource-limited” view of attentional capacity (e.g., Alvarez and Franconeri, 2007); that is, because we can select, individuate and identify any single member of a group of items, our failure to successfully perform these operations simultaneously on all members must derive from the limited resources we have to apply to them. The notion that our limited attentional capacity is caused by limited attentional resources has both implicitly and explicitly informed research for well over a century, effectively constraining questions about attentional function to those concerned with the “resources” that direct attention.

## Competition for representation instead of limited resources

Exactly what is an “attentional resource”? One definition has been “regulatory juice” (Mozer and Sitton, 1998) but exchanging the word “juice” for “resource” is not particularly helpful. Some models of “resources” compare them to a power supply (Kahneman, 1973), such as the amount of gas available to a cooking range. But what would the neural equivalent of “gas” be? Although severe glucose restriction (Ståhle et al., 2011) or oxygen depletion (such as at high altitude; Kramer et al., 1993) can indeed impair cognitive function, low-levels of neither of these metabolites appear to be responsible for the attentional limitations experienced by well-nourished individuals at sea-level. Nor is it the case that attentional limitations are caused by a number of neurons insufficient to represent task-relevant material and cognitive functions. Historically, resources have been sometimes referred to as occurring in “pools”; that is the extent to which these “pools” are available determines the extent to which they may be simultaneously applied to different stimuli or tasks (Wickens, 1984; Wickens et al., 1984). Such theories attempt to separate the “pools” by processing modality, positing that separable sensory (visual, auditory) or cognitive (verbal, spatial) operations will not limit each other because they rely on different regions of neural tissue. At first glance, this may appear to be a sensible heuristic for defining a resource. But consider the vast amount of neural tissue dedicated to processing of visual stimuli; not only are the full visual fields represented many times over, at many spatial scales, but regions broadly specialized for processing certain classes of stimuli (e.g., faces, places, objects) share relatively little neural overlap (Malach et al., 1995; Kanwisher et al., 1997; Epstein and Kanwisher, 1998). And yet, it is still very difficult for us to simultaneously attend to one house and one face (Reddy et al., 2009). What then is the neural basis of this limited processing capacity?

Critically, neither individual neurons nor neural populations operate in isolation. Instead, they have continuous, mutually modulatory interactions; the response of two individual neurons with different receptive fields (RF) that are filled with different stimuli may each be modified by the stimuli outside of their own RF as a result of the neurons' inhibitory or excitatory interactions (e.g., Blakemore and Tobin, 1972; DeAngelis et al., 1992; Desimone and Duncan, 1995; Pelli, 2008). The ability of neural interactions to dramatically influence visual perception has been well-articulated by models of competition for representation (Desimone and Duncan, 1995) and divisive normalization (Reynolds and Heeger, 2009). Specifically, although adjacent visual information may simultaneously fall within the RF of adjacent, but separate cell populations early in visual processing (e.g., V1), the output of the high resolution cells will ultimately converge on a single population of cells at higher level of visual cortex (Gattass et al., 1988), at which point they will interact in a mutually inhibitory manner. For example, two stimuli presented simultaneously within the same RF of a V4 cell evoke less activity than the summed activity of each individual stimulus presented alone (Chelazzi et al., 1998; Reynolds et al., 1999). Similarly, four neighboring stimuli presented simultaneously, and thus capable of competing with each other through mutually inhibitory interactions, evoke lower blood oxygen level dependent (BOLD) responses in V4 than the same stimuli presented sequentially and thus unable to compete (Kastner et al., 1998, 2001; Beck and Kastner, 2005, 2007). Moreover, the magnitude of this difference, which can be viewed as an index of competition, varies as a function of the distance among the stimuli (Kastner et al., 2001) and scales with RF size across visual cortex (Kastner et al., 1998, 2001; Beck and Kastner, 2005, 2007) suggesting that inhibitory interactions among multiple stimuli are strongest when they are likely to fall simultaneously within the same RF. The result of these inter-stimulus interactions is that representations of stimuli presented simultaneously are weaker and coarser than those of stimuli presented alone. Even though these visual stimuli are represented by different cells in both the retina and early levels of visual processing, these representations are mutually modulatory, and thus cannot be said to be “separable.”

What happens when we need the full detail of the visual stimulus to guide behavior? Attention biases the competitive or normalization process in favor of task-relevant material (Desimone and Duncan, 1995; Reynolds and Heeger, 2009), allowing that information to dominate the neural response at the expense of task-irrelevant material. Directing attention to one of multiple stimuli therefore eliminates or reduces the suppressive influences of nearby stimuli, consistent with the idea that selective attention biases the competition among multiple stimuli in favor of the attended stimulus (Moran and Desimone, 1985; Luck et al., 1997; Kastner et al., 1998; Reynolds et al., 1999; Recanzone and Wurtz, 2000). For example, when a monkey directs attention to one of two competing stimuli within a RF, the responses in extrastriate areas V2, V4, and MT are similar to those evoked by that stimulus presented alone (Reynolds et al., 1999; Recanzone and Wurtz, 2000). Similarly, directing attention to one of four neighboring stimuli pushes the extrastriate BOLD activity evoked by simultaneously (potentially competing) stimuli closer to that of the sequentially presented (non-competing) stimuli (Kastner et al., 1998, 2001). These findings suggest that when attention is directed to an item, its representation is protected from the inhibitory influences of unattended stimuli.

Importantly, however, this protection breaks down when attention is directed to multiple competing objects simultaneously. Our work has demonstrated that if attention is directed to multiple items, competitive interactions among them impair their representation. In other words, competition for representation not only limits initial processing, but also our capacity to effectively attend to multiple items (Scalf and Beck, 2010; Scalf et al., 2011a). Consider the case when attention might be directed to simultaneously enhance the representations of multiple competing stimuli. No single stimulus would receive a boost that would enable it to dominate the competitive process; instead, signals from cells whose RFs contained more than one attended item would continue to reflect the contribution of all of the simultaneously attended items. Because attention would be unable to reduce the inhibitory interactions among multiple attended items, the representations of attended items would be weaker than would be the case if a single item received attention. Our previous research has confirmed this prediction (Scalf and Beck, 2010). The receptive field sizes of V4 cells in humans appear to be between 4° and 6° (Kastner et al., 2001); when we asked participants to view multiple adjacent items subtending 2°, we found that BOLD signal evoked in V4 by an attended item was weaker if two neighboring items were also attended rather than ignored. In fact, in another experiment we showed that simultaneously attending to multiple stimuli produced no measurable effect on competitive interactions among attended stimuli (Scalf et al., 2011a). Although attention did enhance V4 BOLD response to the stimulus items relative to when they were unattended, the competitive interactions (assessed by the difference in activity evoked by simultaneous and sequential presentations) were identical for attended and unattended stimuli. Critically, the cost of attending to multiple items simultaneously was specific to conditions in which the items might have required simultaneous representation by a common group of cells (Scalf and Beck, 2010). If the items were presented either sequentially or in opposite visual fields (and thus could be represented either at different times or by different cell populations), then the V4 signal evoked by the cortically isolated item was unaffected by the attentional status of its neighbors. These data demonstrate that attention is less effective at enhancing stimulus representations if it is directed to multiple competing items, suggesting that competitive interactions among stimuli are one cause of humans' limited attentional capacity. Similar arguments can be made regarding multiple salient stimuli, biased via bottom up mechanisms (West et al., 2010).

Although our fMRI data were the first to explicitly link limited attentional capacity to competition for representation (Scalf and Beck, 2010), we were not the first to make such predictions. Nearly two decades of behavioral research support the notion that attentional capacity is functionally expanded when multiply attended items are positioned such that they do not compete for representation. Although not interpreted within the biased competition framework, Sagi and Julez (1985) found that the ability to identify simultaneously presented letters increases with their increasing spatial separation, while Cohen and Ivry (1991) reported that visual search displays containing identical number of items were searched more efficiently when items were widely spaced rather than clumped together. When asked to report two cued letters within a circular 24-item backward masked array, observers are increasingly less accurate as the spatial distance between the cued items is decreased (Bahcall and Kowler, 1999). Similarly, the speed with which observers can make judgments about two cued stimuli (interspersed among fillers) is inversely proportional to the distance between those stimuli; the greater the separation between the attended stimuli, the faster participants are able to respond to them (McCarley et al., 2004, 2007; Mounts and Gavett, 2004; Hilimire et al., 2010). McCarley, Mounts and colleagues explicitly propose that these findings result from the failure of visual cortex to represent multiple attended items that are positioned in a way to compete for representation. Reddy and VanRullen (2007) also appeal to competition in visual cortex to explain why search performance in facial discrimination task improves as the spacing between items increases. The number of moving objects that may be tracked (Alvarez and Franconeri, 2007; Shim et al., 2008; Franconeri et al., 2010) and the number of locations that may be simultaneously monitored for target information (Kristjánsson and Nakayama, 2002; Franconeri et al., 2007) also increases with increasing interstimulus distance, and these data have also recently been interpreted as reflecting competition for representation (Franconeri et al., 2013). Finally, a number of studies have demonstrated that dividing information between the two cerebral hemispheres, or isolating it anatomically from each other in other ways, functionally expands attentional capacity (Luck et al., 1989; Chelazzi et al., 2001; Alvarez and Cavanagh, 2005; Delvenne, 2005; Carlson et al., 2007; Scalf et al., 2007; Torralbo and Beck, 2008; Scalf and Beck, 2010; Alvarez et al., 2012). These behavioral findings are broadly consistent with the notion that attentional “capacity” is functionally limited when items are positioned such that their representations will evoke mutually inhibitory interactions.

Although separation in extrastriate cortex appears to expand capacity, we are not arguing that competitive interactions in extrastriate cortex are the sole source of our limited capacity. Certainly, other limitations exist (e.g., Mecklinger et al., 2003; VanRullen and Koch, 2003; Todd and Marois, 2004; Dux et al., 2006) We suggest, however, that the interactive (competitive) nature of neural representations across even seemingly distinct neural systems may determine the extent to which the information processed by those systems ultimately influences behavior. Although direct evidence of truly competitive interactions between inputs processed by separate neural subsystems is sparse, this framework has been successfully applied to a number of data sets. Neural patterns of activation that are unique to either face or house stimuli despite being anatomically separated, actively suppress one another; this suppression is resolved in favor of the stimulus class to which attention is directed (Reddy et al., 2009). The capacity of visual short term working memory (VSTM) is increased if the to-be encoded items are presented sequentially rather than simultaneously; these changes most likely reflect the improved perceptual representations and decreased attentional demands of the non-competing sequential conditions (Shapiro and Miller, 2011). Neural populations that are responsive to different stimulus modalities (visual, auditory, and tactile) also seem to interact such that visual stimuli dominate response processes unless very specific procedures are used to compensate for the asymmetric excitatory connections between non-visual and visual modalities (for a review see Spence et al., 2012). Decision making and action planning have also been posited to be represented by separate neurons that ultimately compete in a winner-take-all manner to drive a motor response (Cisek, 2006, 2007, 2012). Finally, even the “separable” neural populations of the cerebral hemispheres continuously modulate one another to bias behavior in favor of one representation or another; disruption of these interactions are likely responsible for the effects of neglect and extinction that may follow damage to the parietal lobes (e.g., Rafal et al., 2006; Bays et al., 2011; De Haan et al., 2012). In summary, no matter how great the apparent separation between the neural populations that support behavior, their performance and responses are always informed and modulated by one another. As such, they cannot provide “independent attentional resources” because their activity is in fact always interdependent. We suggest that to the extent that this interdependence is competitive, these interactions will also serve to limit capacity.

## Implications for perceptual load and dilution theories

Our conception of limited resources as competition for representation in the brain has implications for both perceptual load and dilution theories of attention. Both theories purport to explain when and why task-irrelevant information, or so-called distractors are processed. The basic phenomenon that both theories endeavor to explain is the fact that under some task conditions the to-be-ignored items continue to influence behavior, suggesting that they were not actually ignored, whereas under other task conditions the to-be-ignored items appear to be successfully suppressed. Both theories explain the presence or absence of distractor processing by relying heavily on the concept of a limited resource, although they differ in how these resource limitations subsequently affect distractor processing.

Perceptual load theory (Lavie and Tsal, 1994; Lavie, 1995, 2005, 2006, 2010; Lavie et al., 2004) was the first to unify these contrasting results under a single framework. Lavie proposed that it is the perceptual load of the relevant task that determines the extent to which “task-irrelevant,” potentially distracting information is processed. When the perceptual load of the task is low (e.g., set size is small), spare attentional resources obligatorily spill over onto the to-be-ignored items and contribute to their processing. Under high perceptual load (e.g., set size is large), however, attentional resources are exhausted by the relevant task; this leaves no spare capacity for the task-irrelevant items and effectively excludes them from further processing.

Perceptual load theory has received a wealth of behavioral and neural support since its introduction (e.g., Lavie, 1995; Rees et al., 1997; Lavie et al., 2004; Beck and Lavie, 2005; Bahrami et al., 2007; Cartwright-Finch and Lavie, 2007; Forster and Lavie, 2008; Macdonald and Lavie, 2008; Remington et al., 2012). The most well-known evidence in favor of the theory, and the evidence that dilution theory takes issue with, comes from the response competition paradigm. In this paradigm participants search for a target letter and determine its identity (e.g., does the task-relevant display contain X or N?). Critically, in addition to the task-relevant set (i.e., those elements which can potentially be targets), there is a distractor letter that is compatible or incompatible with the target letter (i.e., it either matches or does not match the target). Participants are explicitly instructed to ignore this distractor. Nevertheless, its compatibility with the target can influence reaction times (RTs) to the target, indicating that the distractor's identity was processed. In keeping with perceptual load theory, this influence of the to-be-ignored item is greater in low perceptual load than high perceptual load conditions. In short, the distractor is more fully processed under conditions in which capacity is said to spill over onto the other items in the display.

Dilution theory (Benoni and Tsal, 2010, 2012; Tsal and Benoni, 2010; Wilson et al., 2011), on the other hand, posits that the critical difference between the high and low load displays is not the difficulty of the attentional task *per se* but the presence of multiple heterogeneous stimuli in the high load displays that “dilute” the effect of the distractor. Thus, the reason distractors impact behavior less in high load than in low load conditions is that there are more stimuli competing with the distractor in the high load displays, leaving the distractor a smaller share of the limited processing capacity. Tsal and Benoni (Benoni and Tsal, 2010; Tsal and Benoni, 2010) garner support for their theory by constructing what they call “high dilution/low load” displays; that is, the displays contain many competing letters (high dilution), but the target is made easier to find by virtue of its color or position, such that it qualifies as low load (i.e., detected quickly and accurately). Importantly, despite these displays being “low load” the distractor effects are virtually non-existent; that is, the RTs to targets are comparable regardless of their compatibility with the distractor letter. Tsal and Benoni take this as evidence that dilution rather than load is responsible for the diminished distractor effects.

**Figure 1:**
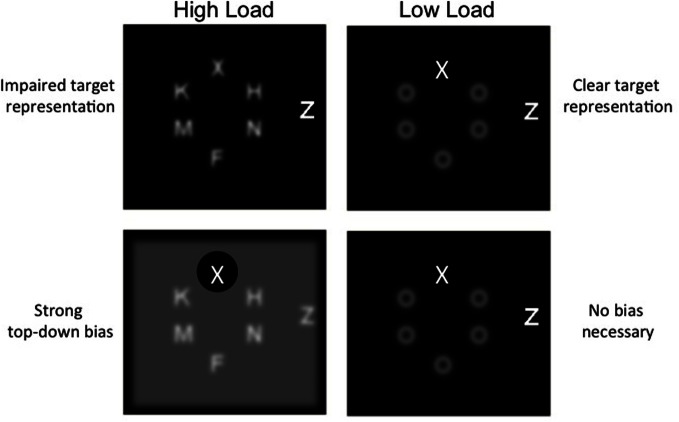
**Schematic depiction of the consequences of competition and top–down bias.** Under high load, competition impairs target representation (**top** panel) and thus a top–down bias is necessary to resolve the target **(bottom)**, which in turn filters out the distractor. Under low load, the target representation is already clear (**top** panel) and thus no bias is needed to perform the task (**bottom** panel).

We propose a third, alternative explanation of the classic perceptual load effect, illustrated graphically in Figure 1 that stems from our formulation of limited resources as competition for neural representation but also shares aspects with both perceptual load and dilution theory (Torralbo and Beck, 2008). As detailed above, all display items compete for representation in visual cortex, and presumably beyond. It consequently follows that high load displays, manipulated via set size, evoke greater competition than low load displays of a smaller set size; that is, rather than being processed independently, multiple nearby stimuli mutually inhibit one another leading to a poorer representation of all stimuli. Low load displays, in contrast, are those that produce less competition among stimuli. This can be achieved with either a smaller set size (which contains fewer competing items) or homogenous non-targets that are distinct from and thus compete less with the target (e.g., pop-out displays; Lavie and Cox, 1997; Beck and Kastner, 2005). Importantly, because the greater competition evoked by high load displays impedes the representation of the target, a strong top-down bias is needed to support its representation. This strong top-down bias then results in the exclusion of the distractor and other non-target items. Under low perceptual load, however, target representation is already clear and top-down bias is unnecessary; in essence, this lack of top-down bias leaves the attentional filter (or window) open, allowing for greater distractor processing.

## Perceptual load and dilution as biased competition

How does our view of perceptual load effects as a consequence of competition and top-down bias map onto perceptual load and dilution theories? Might our conception of the neural processes underlying visual search and distractor effects help distinguish between these two theories? We note that biased competition theory has two components, and both are necessary to explain distractor processing in our view of perceptual load: first, items in a display compete for representation, and second, this competition is resolved in favor of the target (and away from other display items) through a top-down bias.

The concept of dilution maps on well to that of competition. Indeed, Tsal and Benoni (Benoni and Tsal, 2010; Tsal and Benoni, 2010) use similar “competition” language in describing the effects of dilution: when additional letters are added to the high load or high dilution displays “their features compete with those of the incongruent distractor, degrade the quality of its visual representation, thus, substantially reducing the amount of lexical analysis achieved by its corresponding lexical representation (Benoni and Tsal, 2010, p. 1293).” Note that here Tsal and Benoni are even talking about competition at a visual feature level, consistent with known competition effects in visual cortex. The concepts of dilution and competition, however, are also compatible at later lexical and semantic levels.

Tsal and Benoni's description of the exact nature of the competition differs from our own, however. In their view the target, or potential targets, compete specifically with the distractor. In our view, however, the spatial proximity of the potential targets should result in their undergoing much greater competition than occurs between the more widely separated target and distractor (Kastner et al., 2001; Bles et al., 2006). Thus, although the task-relevant display elements may compete with and diminish the representation of the distractor at some level, we argue there is an even more important consequence of visual competition (or dilution); specifically, the target representation may be so impaired by nearby non-targets (as distinct from the response compatible or incompatible distractor) that it is no longer identifiable without selective attention. Thus, accurate identification of the target (as required by the task) requires selective attention (in the form of a top-down bias); without it, the target cannot be resolved within the visual system. According to our view, this concept of a top-down bias is both critical to explaining distractor processing and most clearly distinguishes perceptual load theory from dilution theory.

## The role of a top-down bias

According to our biased competition view of distractor processing, a top-down bias is more directly responsible for the diminished distractor processing in high-load conditions than the competition among stimulus items. It is the application of this bias in favor of the target that results in diminished representation of the distractor. The top-down filter not only enhances the target but also suppresses other stimuli, including the distractor. The notion that enhancement of the target necessarily results in suppression of the other items in the display is fundamental to biased competition theory (Desimone and Duncan, 1995) and divisive inhibition more generally (Reynolds and Heeger, 2009). Because neurons representing individual items have mutually inhibitory connections, an increase in the firing rate of one results in a decrease in the other.

As currently formulated, dilution theory proposes no role for selection of task relevant material. It simply assumes a passive dilution (or competition) among the stimuli. Under high load or high dilution, the distractor receives few resources because its representation is diluted (or weakened) by other items in the display. There is no mechanism to direct resources to task-relevant stimuli; resources are simply depleted by virtue of being spread among too many stimuli. On the other hand, the concept of a *directed* resource is central to perceptual load theory. According to Lavie, high perceptual load tasks “engage full capacity” leaving “no capacity” for task-irrelevant material, whereas low perceptual load tasks fail to exhaust capacity leaving “spare capacity” that “spills over” onto task-irrelevant material (Lavie, 2010, p. 143). In other words, the distinction between task-relevant and task-irrelevant material is critical in determining the extent to which distractors are processed, because this distinction determines the extent to which task-relevant material fully engages a limited processing capacity. This is very similar to our concept of a bias. For us, competition is biased in favor of task-relevant material; ultimately the target item. As in perceptual load theory, this enhancement of task-relevant material occurs at the cost of task-irrelevant material. Unlike perceptual load theory, however, the cost is incurred due to the competitive interactions among elements in the display rather than a depletion of some unspecified “capacity.” We note also that, for us, these competitive interactions occur in visual cortex; that is, the enhancement of the target in visual cortex, by virtue of the inhibitory interactions there, results in a suppression of the other stimuli including the distractor. Perceptual load theory, in contrast, appears to place the source of this capacity, and thus its depletion, in frontoparietal cortex (Lavie and Robertson, 2001; Kelley and Lavie, 2010), although it does acknowledge the interaction between frontoparietal and visual cortices (Remington et al., 2012). In our view, localizing the ultimate source of the competition in visual cortex is more in line with the characterization of this form of load as *perceptual.* As we have already noted, other cognitive capacity limitations, which may play a role in *cognitive* load (De Fockert et al., 2001; Kelley and Lavie, 2010; Lavie, 2010; Carmel et al., 2012), may result from here-to-now unknown competitive interactions among frontoparietal mechanisms.

The need for a strong bias to process high load displays can also explain why “high load” displays that are physically identical to low load displays (Lavie, 1995) can still produce little distractor processing, a set of results that cannot be explained by dilution theory. Such tasks manipulate perceptual load by varying task requirements rather than changing stimuli. For instance, participants are required to detect a conjunction target rather than a target defined by a single feature (Lavie, 1995; Handy and Mangun, 2000; Schwartz et al., 2005; Parks et al., 2011). These conditions require a top-down bias to bind the features together in a conjunction task (Duncan et al., 1997), again resulting in a suppression of the non-targets. In other words, a conjunction search requires a focused selection of the target whereas in feature search the target can be located without engaging a filter, or by leaving the attentional window open.

What is the evidence, first that a bias exists and, second that it enhances the target items at the expense of the task-irrelevant information? There is a large body of evidence showing that when the perceptual load of a task increases it engages frontoparietal mechanisms more strongly (Culham et al., 2001; Pinsk et al., 2004; Schwartz et al., 2005; Scalf and Beck, 2010; Shim et al., 2010; Gillebert et al., 2012; Ohta et al., 2012). Moreover, as proposed by load theory, the fact that a concurrent cognitive task (i.e., higher cognitive load) increases distractor processing presumably reflects the fact that both the selection mechanisms and the concurrent cognitive task draw on the same frontoparietal mechanisms (De Fockert et al., 2001; Lavie et al., 2004; Lavie, 2005, 2010). We add that our view that high load displays require a top-down bias also explains why increasing cognitive load not only increases distractor processing when the perceptual load of the display is low, as proposed by load theory, but also when it is high (Lavie et al., 2004). Top-down frontoparietal mechanisms would be needed in low load to prevent the participant from responding to the well-processed distractor (Lavie, 2005, 2010), but they would also be needed to resolve the target when the perceptual load is high. Thus, any concurrent task that draws on the same frontoparietal mechanisms would increase distractor processing in both cases.

Not only has frontoparietal activity been shown to increase with load, increases in activity in posterior parietal cortex and the frontal eye fields have also been shown to modulate activity in visual cortex (Moore and Armstrong, 2003; Ruff et al., 2006, 2008; Thut et al., 2006; Scalf and Beck, 2010; Scalf et al., 2011a; for review Noudoost et al., 2010). These data are all in keeping with a biased competition model in which attentional control regions in frontoparietal cortex bias activity in visual cortex in favor of an attended stimulus (Desimone and Duncan, 1995; Beck and Kastner, 2009). As already noted, the idea that enhancement of a target occurs at the expense of other stimuli in the display is fundamental to the principles of competitive interactions. If one stimulus is “pushed up” by attention then, by virtue of their competitive/inhibitory connections, other competing stimuli will necessarily be “pulled down.” There are now also numerous neuroimaging studies that find such a push-pull relationship between target and distractor (Somers et al., 1999; Pinsk et al., 2004; Gazzaley et al., 2005; Pestilli and Carrasco, 2005; Hopf et al., 2006). More importantly, the extent of this push-pull relationship (i.e., the difference between activity evoked by task-relevant and task-irrelevant stimuli) is modulated by the perceptual load of the relevant task (Handy et al., 2001; Schwartz et al., 2005; Parks et al., 2011).

## How to define “low load”

Why, according to our biased competition framework of distractor processing, do Tsal and Benoni's “high dilution/low load” displays produce such small distractor effects? Like Lavie, Tsal and Benoni define low load tasks as those that are performed quickly and accurately. According to a biased competition framework, however, it is not the speed with which participants can perform the task that is important, but whether or not a top-down bias is needed to identify the target. The question then becomes whether, under their various “low load” manipulations (Benoni and Tsal, 2010, 2012; Tsal and Benoni, 2010), the representation of the target is clear in visual cortex without further enhancement by selective attention. We believe that if the representation is clear, the attentional window is left open; no bias is then needed to selectively enhance the target at the expense of the distractors, allowing the distractor to be more fully processed. Consistent with this view, Roper et al. (2013) recently showed that the size of the flanker effect increased with increasing search efficiency; that is, those targets that were most likely to be detected in parallel (or with the least amount of serial search) were the most affected by the compatibility of distractors.

We note that implicit in our view of load is the idea that the application of the bias is effortful, and therefore will not occur if the participant can quickly acquire the target without it. This, in a sense, explains why processing of the distractor under low load is, in Lavie's language, obligatory (Lavie, 2005, 2010). Although excluding the distractor may be optimal (i.e., applying a bias even in low load displays), participants are unable or unwilling to do so if the target can be acquired quickly without the bias; after all, participants' primary task is to simply report the target as quickly and as accurately as possible.

What is unclear in Tsal and Benoni's “high dilution/low load” displays is whether or not their “low load” displays actually negate the need for a top-down bias. If they do not, then in our view, they are not “low load” displays. Only if the target is already clearly represented in visual cortex without attention, and thus requires no bias to be identified, would we predict a “low load” processing mode; that is, an open attentional window with no filter. Determining whether Tsal and Benoni's targets are resolved in visual cortex without top-down attention requires knowing how visual cortex responds to their displays under conditions in which the participants' attention is directed elsewhere. For example, we have shown that competition is reduced in visual cortex when the display contains a stimulus that differed in orientation and color from an otherwise homogenous set of stimuli (Beck and Kastner, 2005). This reduction in competition for pop-out displays (relative to a fully heterogeneous stimulus display) was apparent even in V1, and occurred despite the fact that the stimuli were task-irrelevant and subjects were engaged in a demanding central letter detection task. The very early effect of a pop-out stimulus is consistent with single-cell recordings indicating that V1 is sensitive to local feature contrast, specifically when the stimulus differs from a homogenous surround (Knierim and Van Essen, 1992; Kastner et al., 1997, 1999; Nothdurft et al., 1999).

Critically, however, Tsal and Benoni do not use homogenous non-targets and thus our pop-out data do not apply; the presence of heterogeneous letters is what makes their displays high dilution. Instead they use color or position to cue the participant to the target, which speeds RTs and improves accuracy. Are such manipulations sufficient to improve the clarity of the target without attention though? Certainly these manipulations provide cues to guide top-down attention. Participants can use color or relative position to find the target, but if the participants are “using” this information, they may be doing so through a spatially specific top-down bias. Consistent with the idea that subjects are applying a bias, RTs to find the target in the “high dilution/low load” displays of Tsal and Benoni tend to be higher than in the classic low load displays (Benoni and Tsal, 2010, 2012; Tsal and Benoni, 2010).

Of course, color information could, in theory, be used segregate the target in a bottom-up manner. For instance, a unique color might be sufficient to segregate the color singleton from the other letters as early as V1 (Zipser et al., 1996; Li, 1999, 2002), but the question is whether V1 represents the form of the letter sufficiently to support its identification when that letter is surrounded by other letters, even if those letters are of a different color. In other words, it is one thing to say that color makes the target salient in visual cortex, but it is another to say that its form is then necessarily resolvable without attention. As far as we know, this has not been directly assessed in visual cortex.

## Conclusions

We propose a neurally plausible explanation of limited resources in terms of competition for representation. This explanation has consequences for perceptual load and dilution theories. In particular, our biased competition interpretation of the data shares concepts with both theories. The competition component of our explanation is similar to the concept of dilution, the only difference being that the former proposes a specific mechanism for limited capacity behavior whereas the latter relies on the notion of a limited capacity resource. These concepts are easily reconciled, however, if one simply considers competition for representation as the underlying cause of dilution. Our biased competition explanation also shares an important feature with perceptual load theory. Competition can be biased in favor of an item; we argue that this is necessary in high load displays in order to evoke a representation whose resolution is fine enough to support its identification. Importantly then, like perceptual load theory, biased competition theory predicts that “resources” are directed toward the target at the expense of the non-targets and distractors. Dilution theory, in contrast, provides no such distinction.

In short, because our biased competition framework incorporates aspects of both dilution and perceptual load, we see it as a hybrid of both theories that incorporates known neural mechanisms. We believe dilution occurs and is responsible in part for the presence or absence of distractor processing. We also believe, however, that this cannot be the full explanation of the “perceptual load effect.” Instead, a mechanism for directing attention and filtering out task-irrelevant stimuli must also play a role in distractor processing.

Finally, although our explanation shares similarities with both perceptual load and dilution theories, we see it as an improvement on both because it draws on known neural mechanisms and provides a neurally plausible alternative to the concept of a fixed capacity resource. Admittedly, the notion of a capacity or resource provides an intuitive metaphor with considerable predictive validity for explaining limited capacity behavior; it is unlikely, however, to be accurate at a neural level. In our view, the actual implementation of the “limited capacity” more likely reflects the interplay of competition for representation and top-down biases invoked to resolve the competition in favor of the target.

### Conflict of interest statement

The authors declare that the research was conducted in the absence of any commercial or financial relationships that could be construed as a potential conflict of interest.
